# “I feel so stupid because I can’t give a proper answer…” How older adults describe chronic pain: a qualitative study

**DOI:** 10.1186/1471-2318-12-78

**Published:** 2012-12-31

**Authors:** Amanda Clarke, Geraldine Anthony, Denise Gray, Derek Jones, Paul McNamee, Patricia Schofield, Blair H Smith, Denis Martin

**Affiliations:** 1 Faculty of Health and Life Sciences, Coach Lane Campus, Northumbria University, Benton, Newcastle upon Tyne, NE7 7XA, USA; 2 University of Dundee, Medical Research Institute, Division of Population Health Sciences, Dundee, UK; 3 University of Aberdeen, School of Medicine, Aberdeen, UK; 4 University of Greenwich, School of Health and Social Care, Greenwich, UK; 5 University of Teesside, School of Health and Social Care, Teesside, UK

**Keywords:** Older adults, Ageing, Qualitative research, Stories, Metaphors, Chronic pain, Community

## Abstract

**Background:**

Over 50% of older adults experience chronic pain. Poorly managed pain threatens independent functioning, limits social activities and detrimentally affects emotional wellbeing. Yet, chronic pain is not fully understood from older adults’ perspectives; subsequently, pain management in later life is not necessarily based on their priorities or needs. This paper reports a qualitative exploration of older adults’ accounts of living with chronic pain, focusing on how they describe pain, with a view to informing approaches to its assessment.

**Methods:**

Cognitively intact men and women aged over sixty-five who lived in the community opted into the study through responding to advertisements in the media and via contacts with groups and organisations in North-East Scotland. Interviews were transcribed and thematically analysed using a framework approach.

**Results:**

Qualitative individual interviews and one group interview were undertaken with 23 older adults. Following analysis, the following main themes emerged: diversity in conceptualising pain using a simple numerical score; personalising the meaning of pain by way of stories, similes and metaphors; and, contextualising pain in relation to its impact on activities.

**Conclusions:**

The importance of attending to individuals’ stories as a meaningful way of describing pain for older adults is highlighted, suggesting that a narrative approach, as recommended and researched in other areas of medicine, may usefully be applied in pain assessment for older adults. Along with the judicious use of numerical tools, this requires innovative methods to elicit verbal accounts, such as using similes and metaphors to help older adults describe and discuss their experience, and contextualising the effects of pain on activities that are important to them.

## Background

Chronic pain affects more than 50% of community-dwelling older adults and there is an age-related increase in individuals up to 80 years old [1]. The prevalence of musculoskeletal pain at most sites increases with age, including the back, knees, hips and other joints [2,3] and neuropathic pain is also more common in older adults [4]. Chronic pain in later life can have a profound impact on the individual’s quality of life, reducing social networks and physical activities, independent functioning and emotional wellbeing [5-9]. The high figures for pain in older adults may even represent underestimates, as a result of under-recognition and under-reporting of pain which, in turn, leads to under-treatment [10]. There are wide misconceptions about pain and its management in later life; for example, it is suggested that some clinicians see pain as an inherent consequence of ageing [10] although research does not substantiate this view [1]. Regarding older adults with pain, the few studies focusing on their perspective suggest a tendency to expect pain, a strong desire to be a “good patient,” and a reluctance to complain [10-12]. Interviews with older adults with severe osteoarthritis revealed a degree of resignation about pain [13], whilst a narrative study found that stereotypical views of older adults as constantly talking about pain and illness affected their confidence in expressing feelings about their pain [14]. The association of older age and pain [13,14] may also obscure the effort that is involved in living with chronic pain [15]. While some older adults may seem to deny the presence of pain, by preferring to use words such as “comfort”, “hurting”, or “aching” rather than “pain”[16]. Absence of report of pain and its effects should not, therefore, be taken as absence of pain and its effects. It appears that mistaken beliefs about ageing and pain persist amongst some older adults and some health professionals, and that these may influence the way pain is reported, described and discussed by older adults.

The aim of this paper is to gain insight into how older adults with chronic non-cancer pain describe their pain, with a view to informing professional approaches to its assessment. We draw on qualitative data from the first phase of a mixed methods study: Engaging with Older People and their Carers to develop interventions for the self-management of Chronic Pain (EOPIC) [17]. We focus here on findings directly relating to participants’ responses to questions asking them to describe their pain, numerically and verbally.

## Methods

### Setting and Sample

We recruited participants from the community through advertising widely in the media (radio, television and newspapers) and via contacts with groups and organisations in North-East Scotland (charities, council groups, faith groups, carers groups, Black and Minority Ethnic groups, luncheon clubs). Despite rigorous efforts to recruit individuals from Black and Minority Ethnic (BME) groups, the only BME individuals who opted into the study were from a Chinese luncheon club. Potential participants contacted the Research Associate by phone or email if they wished to participate in the study, she described the study to them and, if they were still interested, they were sent an introductory letter, information sheet and consent form. We included all those who volunteered to participate if they were aged 65 or over, had self-reported non-cancer chronic pain, and were cognitively able to give informed consent.

### Design

A qualitative approach was chosen since relatively little is understood about chronic pain from the perspective of older adults living in the community. Qualitative interviews were conducted in order to obtain rich (in a conversational sense) data about participants’ descriptions of pain contextualised in their own experiences [18]. We aimed to undertake two interviews with each participant using a topic guide based on our review of the literature and discussions with our advisory group: first, to ask open-ended questions about day-to-day living with pain; and second, to ask questions focused on the individual in order to follow-up and clarify emerging themes; this gave participants the opportunity to elaborate on their answers in the first interview [18].

### Procedure and Data Collection

Out of the 23 participants who volunteered to participate in the study, 14 agreed to be interviewed twice, the seven Chinese participants declined to participate further and two men were unable to participate further due to ill-health. Second interviews were undertaken four to six weeks after the first. Individual interviews were undertaken by two researchers (both of whom had experience of conducting qualitative interviews) in participants’ homes with all but the Chinese participants who preferred to be interviewed together as a group at a luncheon club. Although this diverted from our original intention to conduct individual interviews, it was in keeping with qualitative approaches which aim to respond flexibly and sensitively to the needs of participants in order to capture a range of experiences and views [19]. A worker at the luncheon club, who was bilingual, played an intermediary role as interpreter during the interview when needed. In a group interview, respondents are questioned simultaneously for individual responses rather than the dynamic, interactive responses of a focus group [19]. So, although not as in-depth as individual interviews, the focus is still on individual, rather than collective responses [19]. Each interview lasted approximately an hour, was digitally recorded with permission, and transcribed.

Interviews began by asking participants to, ‘Tell me about your pain, please?’ and, ‘What is it like to live with pain?’ Subsequent questions aimed to elicit descriptions of pain: ‘How would you describe the pain?’ and, ‘Have there been other painful episodes in your life?’ The themes described below arose from participants’ answers to these three questions, together with their responses to being asked to rate their pain. As part of the general information collected for comparison purposes in the broader study, participants were asked to rate the severity of their pain at its worst and on a general, day-to-day basis on a simple 0-10 rating scale.

### Analysis

The interview recordings were transcribed by a university-based transcriptionist. The Research Associate compared the transcripts with the digital-recordings to ensure accuracy of the transcriptions which were anonymised to ensure confidentiality. Interviews were managed with a qualitative data software programme (QSR NVivo version 8; QSR International, Doncaster, Australia). Data collection and analysis were concurrent and analysis iterative; this allowed emerging themes to be explored in consecutive interviews. To facilitate rigorous data analysis, we were guided by the principles of Framework Analysis, a matrix-based method developed for policy research but increasingly used in applied health services research [20,21]_._ This involves a structured process of sifting, charting and sorting the transcribed data according to key themes [21]. The transcripts were read and preliminary themes were applied to the text independently by two researchers, who then debated and agreed a common framework which, in turn, was discussed with all the multi-disciplinary team of researchers and the advisory group (which included older adults with chronic pain).

### Trustworthiness of data

Aware that researchers’ attitudes may influence design, data collection, and analysis of qualitative studies, all the research team and advisory group were consulted to enhance balanced interpretation and reliable analysis of the data [22,23]. Divergent interpretations were discussed until a single common set of themes was agreed. The resulting thematic framework was then systematically applied to each transcript, whilst being open to incorporating emergent themes raised by participants. We moved back and forth between the full interview transcripts and the thematic framework to ensure interpretations were valid and contextualised in participants’ broader accounts.

### Ethical considerations

The study was approved by the North of Scotland Research Ethics Committee (09/SO802/93). Prior to each interview, the interviewer explained the study again, gave participants the opportunity to ask questions and reminded them about the voluntary nature of their participation. Verbal and written informed consent was obtained from all participants before their first and second interview. In the case of Chinese participants, although they appeared to understand spoken and written English, they reported less confidence about speaking English and a bilingual worker at the luncheon club helped to explain questions they had about the research before they made a decision to take part.

## Results

Twenty-three men and women agreed to participate in the study. Demographic data were collected at the start of each interview. Participants’ ages ranged from 66 to 89 years (median =73 years) of whom 16 were female. Sixteen participants were Caucasian, the remainder Chinese. The local population is about 98% Caucasian. All participants were retired, with the exception of one man who worked full-time, 15 lived alone and 8 lived with their spouses. Health conditions were self-reported and participants most commonly described their condition as arthritis. All reported chronic pain with ten participants describing multiple sources of pain experienced over 5 to 20 years.

We observed three clear themes from analysis of the data with respect to the question: how do older adults describe chronic pain? These were: diversity in conceptualising pain using a simple numerical score; personalising the meaning of pain by way of storytelling and metaphors; and, contextualising pain in relation to its impact on everyday activities. An illustrative range of quotes from different participants (here ascribed a code with F=female, M=male) was chosen that most succinctly captured a theme across the interviews.

### Diversity in conceptualising pain using a simple numerical score

We found amongst individuals and circumstances that, sometimes, a simple numerical rating scale was feasible to complete verbally and regarded as appropriate, while sometimes, it was considered difficult, unhelpful or too simplistic. When asked by the interviewer, ‘Can you please rate your pain between 0 and 10 where zero is no pain and ten is the worst pain you can imagine?’ in general, participants appeared confident in rating their highest severity of pain, ‘*Ten without a doubt, it’s excruciatingly sore.’* (Caucasian Female 8 aged 86 with knee joint pain). In contrast, some participants were more hesitant when asked to give a general or day-to-day rating to their pain, as this exchange between the interviewer and one woman shows; the participant never actually provided a general pain rating:

"‘Can you please rate your pain between 0 and 10 where zero is no pain and ten is the worst pain you can imagine? (Interviewer) Oh, eight to ten."

"And on a daily basis, are you able to give it a rating between 0-10? (Interviewer)"

"It’s all right when I’m in bed, as soon as you get up in the morning it starts again. It more or less remains until it fades itself away, you know, towards the end of a fortnight.’ (Caucasian Female 4, aged 79 with back pain)."

In response to the same question, the following extract shows how, initially, another woman struggled to rate her pain but did so after prompting by the interviewer:

"‘It’s worse in the evening. In the morning I take a cup of coffee and it keeps me going, but I think from five o’clock onwards in an evening, it’s worst."

"And how would you rate your pain on that scale? (Interviewer)"

"Well, the average day is bad [laughs] the pain doesn’t go away. But the time of day in which it’s worst, is between five o’clock and ten o’clock in the evening, even up to eleven o’clock."

"Okay. And could you say if zero was no pain and ten was the most…(Interviewer)."

"There’s no time when there’s no pain. One is very light pain…Two is a wee bit… one, two, three, four, five, up to five o’clock is bearable, but it’s light at first provided I take my coffee. Then it goes steadily up and also saps my energy.’ (Caucasian Female 1, aged 78 with joint pain."

Amongst those who reported difficulty rating their pain, when asked why, Caucasian participants talked about the perceived inadequacy of a single rating to describe pain that fluctuated over time, day-by-day or hour-by-hour:

"So I wondered… what is it about putting a number to it? What is difficult? (Interviewer)."

"Because I think it’s never the same for a long length of time. Like, my leg can be devilling and sore when I’m sitting here. It’ll maybe be like that for another couple of hours, you know… so I think that’s why you can’t get a proper rating to it all. (Caucasian Female 13, aged 65 with back and leg pain)."

In contrast, all of the Chinese participants unhesitatingly ascribed a number to their pain, explaining that since English was their second language, it was easier to give a number than a verbal description; however, in their home country, they reflected that this would not be problematic:

"[in the UK] when we suffer pain we go to the GP okay, but even if we are in pain, really in pain, we cannot describe it to the GP because of the language barrier. But in Hong Kong if we are in pain we just tell the GP, ‘I want this arm off, I really want it off!’ [laughs] (Chinese Male 4, aged 73 with joint and muscle pain)."

For the Caucasians, for whom language was not a problem, there appeared to be a general preference to describe pain verbally rather than numerically: even so, one man spontaneously used a numerical scale - the Richter Scale - to rate his pain in response to a question about what helped to relieve his pain:

"‘Aye, it’s there … to put it on the Richter Scale nothing to five, if I sit down and I’m relaxed it’s one, if I get up and walk it’s five’. (Caucasian Male 18, aged 75 with joint pain)."

### Personalising the meaning of pain by way of stories, similes and metaphors

When asked the question, ‘Tell me about your pain?’ Caucasian participants were keen to do so and used descriptors such as *nagging, sore, uncomfortable, absolute agony and unbearable.* Rather than using isolated words to describe their pain, however, participants were inclined to construct a ‘story’ around pain and sometimes used similes and metaphors; that is, they spoke about their pain in terms which were suggestive of something else [24]: for example, likening their pain to ‘two bones rubbing together,’ and the sensation of ‘running cold water.’ These metaphors tended to be vivid and powerfully conveyed the severity of their pain: 

"Well it’s excruciatingly sore. I thought it was torture and I was thinking of Bloody Mary in the sixteenth century, and I thought in this day and age we shouldn’t have to suffer like this. (Caucasian Female 8, aged 86 with knee pain)."

Indeed, Chinese participants described pain at its worst as being synonymous with death, stating, ‘it’s worse than death’, or ‘death would be preferable to pain’.

As with the numerical scale, a few participants said that they found it hard to describe their pain in words and, when specifically asked why, again replied that it was to do with its variability and unpredictability; pain was ‘ever present’ and ‘worse at some times than others.’ When asked to describe his pain, one man replied, ‘I feel so stupid because I can’t give a proper answer.’ He went on to express his embarrassment about this:

"‘I can’t really explain to you how embarrassed I feel because really there isn’t words to explain…"

"Why would you feel embarrassed about it? It’s an interesting term, embarrassment (Interviewer)."

"That I don’t have the vocabulary and I haven’t got the medical vocabulary. I don’t have the jargon to explain what I'm feeling.’ (Caucasian Male 9, aged 68 with foot pain)."

Given space and time in the interview, however, he found that the use of a metaphor helped him to do so:

"‘It’s there ever present, it’s there right now and it’s worse at some times than others. It’s a very difficult thing to describe to you. I used to work in the dairy industry and we used refrigeration quite a lot, and if you listen to refrigeration going through pipes, that’s exactly the same feeling as I have in my feet; it’s a sort of a bubbling.’ (Caucasian Male 9, aged 68 with foot pain)."

Comparing chronic pain with previous personal incidences of acute pain by way of storytelling also seemed to be a useful way to set current painful experiences in context:

"‘Well, the sorest pain I ever got was playing football in Glasgow on an icy morning, and in these days the ball was leather…, the ball hit the ground and it came up to me and it hit me right in the short and curlies, and that was really painful. Well, when it’s sore it can reach that.’ (Caucasian Male 18, aged 75 with joint pain)."

### Contextualising pain in relation to impact on everyday activities

In the narrative response, the ‘story’ often concerned how chronic pain restricted or completely prevented normal function and activity, such as walking the dog or going to the Post Office. How pain affected physical or social activity was often used as a means of indicating its severity and impact; jobs around the house and garden that were once taken for granted were now ‘challenging,’ requiring adaptations:

"‘Some things take much longer than before. I can only do limited gardening, you know, like cut the grass or that and I do it in wee stints.’ (Caucasian Male 18, aged 75 with joint pain."

Again, the variability of the pain throughout the day was emphasised in relation to the impact on activities:

"It’s always uncomfortable, but sometimes… you know, I know I’ve to be very careful with things but I suppose I’ve lived with it so long I can just come down the stairs and get on and do my general things, and as the day goes on it gets worse. As the day goes on it does get worse, and it does depend what I’m doing, and I mean, I love the garden, I enjoy working in the garden but there’s just a certain amount I can do in the garden. And even John will be saying to me if I’m out there for a while ‘you better get inside or you’ll be suffering’ because then at night I’m just sitting in absolute agony and, you know, I’ve taken all the painkillers I can take. (Caucasian Female 13, aged 66 with back and leg pain)."

Participants also discussed at length the impact of pain on limiting social activities such as shopping, bowling and visiting friends, despite the embarrassment this caused:

"‘I belonged to three walking clubs at one time and would still like to go out walking, but I end up keeping everyone back because I’m so slow and I’m embarrassed by it, and I’m very sore.’ (Caucasian Female 2, aged 78 with hip pain)."

The impact of pain on social activities was sometimes perceived to have a knock on effect on people’s friendships:

"‘Och well I can’t walk, I can’t go shopping in malls and things like that. I'm no use to my friends because they're all active, everybody wants to go for a walk and I can't.’ (Caucasian Female 1, aged 72 with back and knee pain)."

The everyday, taken-for-granted tasks, that pain restricted such as fastening clothes and putting on make-up were emphasised:

"‘I can't put on my bracelet and this blouse has tiny buttons but it was out yesterday and I just put it on again today and I have pearls and I have a job linking them. I can get them in and then I sit and sometimes just sheer determination I try and try and try to put it on, but it’s just hopeless. I always had nail varnish on…’ (Caucasian Female 8, aged 86 with joint pain at multiple sites)."

Giving specific details about the impact on activities also revealed the incremental deterioration of their pain:

"‘The fall injured my knee worse, because if you fall you fall onto your knees and I used to walk - every time I went out I walked. I still do walk, I walk to Debenhams on a Sunday, meet a sister-in-law there for a coffee and I can walk to the Blind Centre in Cromwell Street, down there, cross Clifton, down Greenwood Place, past the hospital and you’re there. So that gives you the length of journey. But I did that all the time; I don’t do it so much now. Last two years have taken its toll on me.’ (Caucasian Female 1, aged 81 with joint pain)."

## Discussion

Three themes relating to descriptions of chronic pain emerged from our analysis: diversity in conceptualising pain using a simple numerical score; personalising the meaning of pain by way of stories, similes and metaphors; and, contextualising pain in relation to its impact on everyday activities. As in our study, others have reported that the variation and unpredictability of pain makes it hard to rate numerically, and not just amongst the older population [25,26]. There is a viewpoint that questions the meaningfulness and value of ascribing a number to complex experiences such as pain [27]. Nevertheless, simple scales, including the numerical rating scale used in this study, are widely recommended and have provided much useful information [28,29]. The findings from our study suggest that the simple numerical scale used here may not provide the scope for some older adults to adequately convey some of the features, variation and potential outcomes that they see as important [26]. There are a number of pain assessment scales that are purported to be effective when assessing pain in older adults such as numerical rating scales or verbal descriptors (BPS/BGS Guidelines for the Assessment of Pain in Older Adults 2007 [30]. Nonetheless, it is acknowledged that such scales only measure one aspect of pain which is intensity: it is essential to adopt a holistic, multi-dimensional approach to assessment to capture more of the complexity of pain; such as using a variety of terms synonymous with pain to screen older adults [30]. Cicely Saunders introduced the concept of ‘total pain’ which places emphasis on the person and his or her experience of illness; recognising that their pain might arise from physical, psychological, spiritual or social distress, rather than simply referring to the body and its manifestations of disease [31]. An individual’s concern, for example, about delays in diagnosis may impact upon their physical experience of pain. Efforts should therefore be made to provide scope for storied accounts that have the potential to provide contextual information that is more personally meaningful; inadequate exploration of pain is likely to be detrimental to comprehensive assessment of needs and thus poor management of symptoms and evaluation of treatment [32-35].

Our findings demonstrate the value of describing pain verbally in the context of the individual’s life [13,14]. Older adults were inclined to construct a narrative around their pain, even in response to ‘closed’ questions, and used this as the basis for much of their interviews, to give their pain some context and meaning. This is a similar finding to that of Gooberman-Hill and colleagues [26], who used a different method, the ‘questerviews’ technique, to elicit older adults’ descriptions and experiences of joint pain in the context of standard self-assessment questionnaires (which included the Hip Disability and Osteoarthritis Outcome Score). They concluded that the assessment or measurement of pain should take into account the importance of pain experience, as well as severity, through use of patient narrative accounts. In our study, participants also related their pain to physical or social activity as a means of indicating its severity and impact; for example, their ability to walk, on social activities and/or having to adapt these activities. This suggests that ‘measuring’ the impact of pain against activities that are important to the older person may be another approach to gauging the effect of pain management; for example, by asking, following treatment, if they are able to walk any further with their dog, or about any increased social activity. Sometimes, the use of similes and metaphors provided a useful vehicle to overcome initial difficulties in describing pain and was used to indicate its severity.

Current UK policy states that practitioners should work in partnership with older adults in order that early intervention, assessment and management of chronic pain is founded on the older individual’s needs and wishes [36]. This can only be achieved if we are willing to listen to older adults’ accounts of chronic pain. In this respect, chronic pain medicine may learn from palliative medicine, where, attending to the central message of individuals’ stories in terms of improving understanding of their total needs has a long tradition [37].

In practice, this requires allocation of time and a willingness to listen. Dillon et al [38] used a post-test only, randomised, double-blind experiment to test how the phrasing of health practitioners’ pain questions affected the pain information provided by community-living older adults with chronic osteoarthritis pain. They found that phrasing of the practitioners’ pain questions significantly impacted the amount of important pain information provided by older adults. Use of an open-ended request ‘tell me about your pain, aches, soreness or discomfort’ resulted in significantly more pain information than the closed-end question, ‘what would you rate your pain, aches, soreness or discomfort on a 0 to 10 scale, with 0 no pain, and 10 the worst pain possible?’ or the more social desirability-biased question, ‘how are you feeling?’ Social desirability refers to individuals responding in ways that are perceived to be the most socially acceptable rather than reflecting how they actually feel. Dillon and colleagues [38] concluded that practitioners should routinely ask open-ended pain questions when initiating pain assessments in order to provide a more tailored pain management approach for older adults. They also suggest that older adults should be helped to develop the most effective ways to communicate their pain to practitioners.

In terms of research approaches, seeking a more holistic assessment of pain can be more problematic in studies using quantitative designs; however, designs that include nested qualitative studies can provide this information. Methods that incorporate quantitative and qualitative approaches, such as the Day Reconstruction Method [39,40] which uses structured diaries to collect data, may be worth further investigation and, as new technologies emerge to enable easier capture of rich objective and subjective data, such approaches may become more feasible in the future.

In common with much qualitative work, our findings should be treated as illustrative rather than statistically representative so there may be more issues in this field that have not emerged. Since we recruited through advertisement, it might be expected that people responded who wanted to talk about their pain and who did not have a straight forward answer to questions concerning their condition. The geographical location - North East Scotland - meant that possible influences and interplay of factors such as gender, ethnicity, socio-economic circumstances or sites of pain, could not be investigated in-depth and requires further study. The findings from the Chinese participants in particular, raised important questions about the assessment of people from ethnic backgrounds and whose first language is not English. However, a limitation is that the Chinese participants chose to be interviewed in a group, only once, and, at times, needed the help of an interpreter to clarify our questions and help them express their answers. This had an effect on the depth of data obtained; their answers tended to be short and probing to expand answers was difficult. It may therefore be seen as unsurprising that Chinese participants were able to ascribe a number to their pain, rather than detailed, verbal descriptions. In addition, it is possible that less confident participants may have deferred to the views of the more vocal members, although we did encourage everyone to speak in turn [41]. This line of enquiry needs further investigation since health care professionals may need to use different approaches with different minority groups [42]. The numerical scale was restricted to a simple numerical rating scale and so the findings do not necessarily relate to other measurement tools: that would be another useful extension of this study.

## Conclusions

Our findings highlight the importance of narrative as a meaningful way of describing pain for older adults. The findings of the study raise questions as to how best narrative medicine, as recommended and researched in other areas of medicine [37,43,44], might be applied in pain assessment and management for older adults. Along with the judicious use of recommended numerical tools, this may require innovative methods to elicit verbal accounts. The findings demonstrate the potential for encouraging the use of similes and metaphors to help older adults to describe and discuss their experience, and also the potential value of contextualising the effects of pain on activities that are important to the older person. This will be clinically useful in assessing chronic pain and evaluating its management in this group of people.

### Key points

● As a multi-dimensional and conceptual experience, chronic pain requires a holistic approach to assessment.

● A narrative approach helps facilitate the older person’s conceptualisation of pain and the impact of pain on his or her life.

● Narrative methods of assessing pain and its impact, such as asking the older person about the effect of pain on activities which are important to him or her and encouraging the use of storytelling, similes and metaphors, should be considered for routine practice.

## Competing interests

The authors declare that there are no competing interests.

## Authors’ contributions

AC, DG, DJ, PM, PS, BS and DM all made substantial contributions to the conception and design of the study. DG carried out most of the data collection and analysis of data. AC helped undertake the data collection and analysis and drafted the manuscript. All authors contributed to data analysis and interpretation, critically commented on, and approved the final manuscript.

## Authors’ information

AC: PhD, MA, BA (Hons), PgDipEd (Health), RGN

GA: BSc, RM, RN

DG: MSc, BSc (Hons)

DJ: PhD, BA (Hons) Dip COT

PM: PhD, MSc, MA

PS: PhD, PGDipEd, DipN, RGN

BS: MBChB, MEd, MD, FRCGP, FHEA, FRCP Edin.

DM: DPhil, MSc Applied Statistics, BSc (Hons) Physiotherapy

## Pre-publication history

The pre-publication history for this paper can be accessed here:

http://www.biomedcentral.com/1471-2318/12/78/prepub
